# Utilization of cardiopulmonary bypass for resection of a massive left retrotracheal thyroid goiter: a case report and review of literature at a tertiary care hospital in Saudi Arabia

**DOI:** 10.1093/jscr/rjag214

**Published:** 2026-06-14

**Authors:** Maya A Alharbi, Raghad A Ghazzawi, Ahmed A Jamjoom, John C Heaphy

**Affiliations:** Medical Student, King Abdulaziz University Hospital, 21589, Jeddah, Makkah province, Saudi Arabia; Department of Otolaryngology–Head & Neck Surgery, King Faisal Specialist Hospital and Research Centre, 23431, Al Rawdah District, Jeddah, Makkah province, Saudi Arabia; Department of Cardiovascular Surgery Section, King Faisal Specialist Hospital and Research Centre 23431, Al Rawdah District, Jeddah, Makkah province, Saudi Arabia; Department of Otolaryngology–Head & Neck Surgery, King Faisal Specialist Hospital and Research Centre, 23431, Al Rawdah District, Jeddah, Makkah province, Saudi Arabia

**Keywords:** cardiopulmonary bypass, median sternotomy, posterior mediastinal thyroid goiter, thyroidectomy

## Abstract

Substernal goiter consists of thyroid tissue extending into the thoracic cavity and is more common in females. Total thyroidectomy is standard, though some cases require advanced approaches. Cardiopulmonary bypass (CPB) may be considered in selected cases for hemodynamic stability. We aimed to describe a rare case of retrotracheal thyroid goiter managed using CPB and review reported surgical approaches. A 39-year-old male presented with hoarseness and palpitations due to a left-sided retrotracheal thyroid goiter. Initial cervical access was unsuccessful, requiring median sternotomy with CPB for safe resection. Histopathology confirmed a benign follicular nodule, and the postoperative course was uneventful. CPB can be a valuable adjunct in complex retrotracheal substernal goiters, highlighting the importance of individualized surgical planning and a multidisciplinary approach.

## Introduction

A substernal goiter is thyroid tissue extending into the thoracic cavity, more common in females, with a female-to-male ratio of ~4:1 [1]. Growth is slow and often asymptomatic, detected incidentally on chest radiography (X-ray), computed tomography (CT), or magnetic resonance imaging (MRI). Symptoms result from compression, most commonly exertional dyspnea, choking, cough, and stridor [1, 2]. Initial evaluation includes thyroid-stimulating hormone (TSH), with total or free thyroxine (T4) and triiodothyronine (T3). CT is preferred, with MRI as an alternative, and X-ray may show an upper mediastinal mass with tracheal deviation [2]. Surgery remains the definitive treatment, most commonly total thyroidectomy. Approximately 17% require partial sternotomy or thoracotomy. According to the Mercante classification, grade II and III goiters more often require extra-cervical access [3]. We report a case of a 39-year-old male with a left-sided retrotracheal thyroid goiter managed through a collar incision, median sternotomy and cardiopulmonary bypass (CPB).

## Case presentation

**Figure 1:**
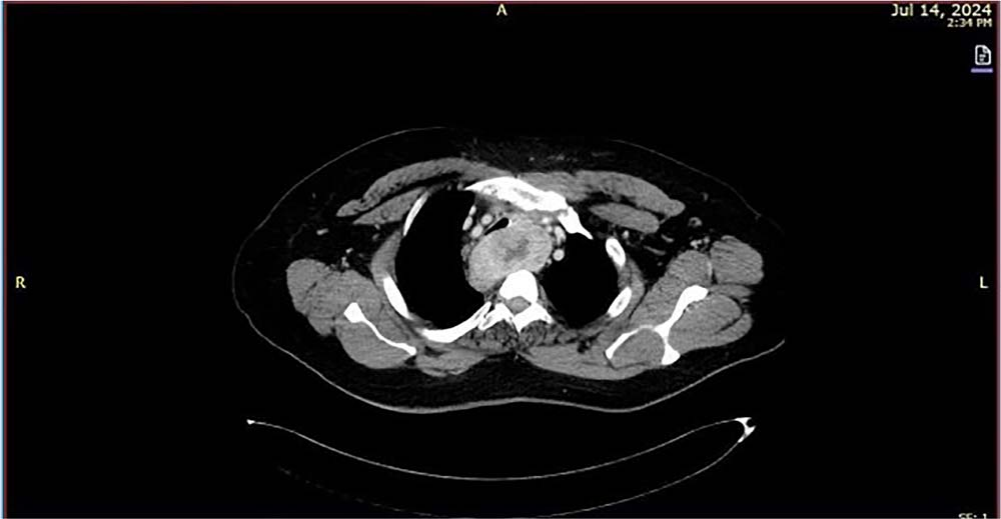
Transverse CT image showing a heterogeneous posterior mediastinal mass with central necrosis.

**Figure 2:**
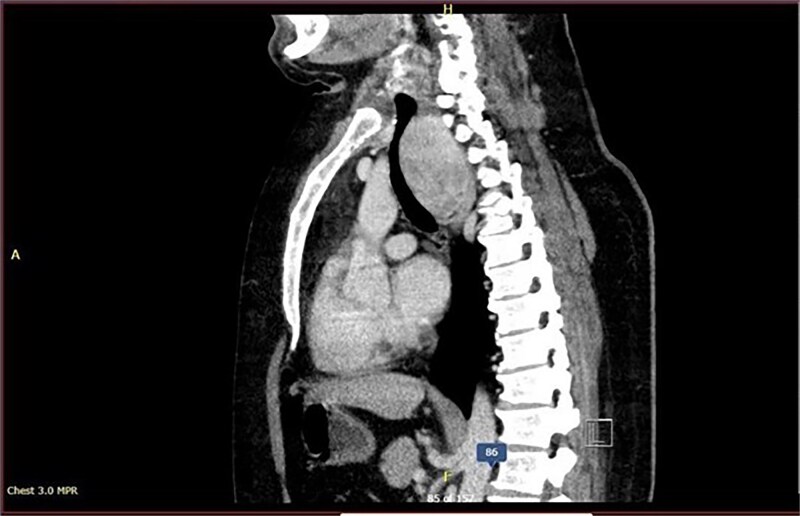
Sagittal CT image demonstrate a posterior mediastinal mass with significant compression and displacement of adjacent structures.

**Figure 3:**
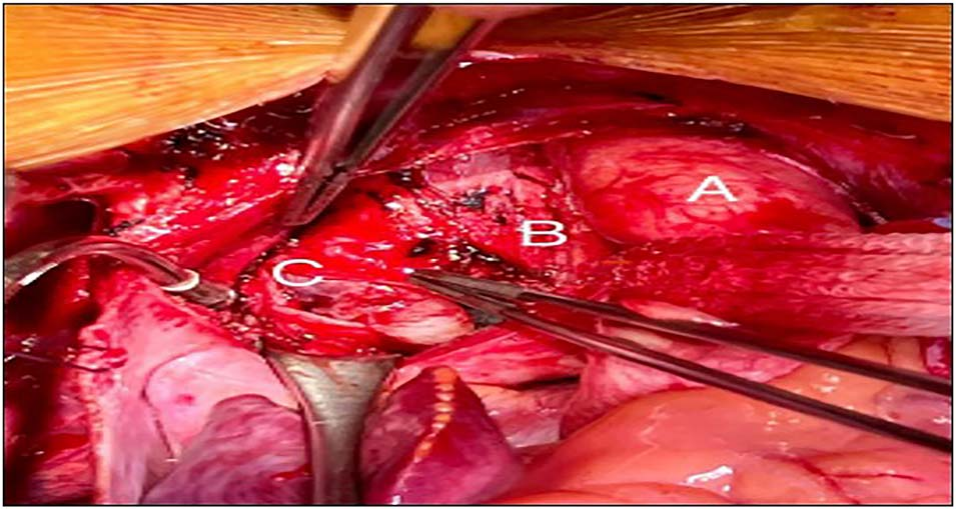
An intraoperative picture demonstrate goiter (C), trachea (B), and aorta (A).

A 39-year-old male with obstructive sleep apnea and hypertension was referred to ear, nose, and throat (ENT) for a left thyroid goiter with retrosternal extension. He reported 4 years of noisy breathing, progressive dysphagia, shortness of breath when bending forward, episodic sleep apnea, intermittent morning hoarseness worsened by upper respiratory infections and exertion, and palpitations in the left lateral decubitus position. Symptoms worsened over 6 months. He denied hypo- or hyperthyroid symptoms. Past surgical history included adenotonsillectomy; no prolonged intubation or radiation exposure. He was a non-smoker with a family history of hypothyroidism. On examination, body mass index was 36.5 with a short neck, no palpable masses or lymphadenopathy. Flexible nasopharyngolaryngoscopy showed restricted left vocal cord mobility. Thyroid function tests: TSH 0.69, free T4 25.4, T3 4.8. Contrast-enhanced CT revealed a 7 × 4.8 × 7.4 cm heterogeneous posterior mediastinal mass with central necrosis, compressing airway and displacing mediastinal structures, arising from the inferior left thyroid lobe. Multiple small cervical lymph nodes were noted. Ultrasound-guided biopsy suggested benign follicular nodular disease (Figs 1 and 2). An initial cervical collar incision failed to provide adequate access, necessitating median sternotomy. Intraoperatively, the trachea was found deep beneath the ascending aorta. Manipulation of the deeply seated goiter resulted in hemodynamic instability, prompting initiation of CPB. Following heparinization and cannulation, full-flow bypass was established, ventilation was paused, and the trachea was mobilized to allow safe resection of the retrosternal goiter. The patient was subsequently weaned from bypass without complication. Postoperatively, recovery was uneventful apart from transient hoarseness. Histopathology confirmed follicular nodular thyroid disease with no malignancy. At 6-month follow-up, hoarseness had significantly improved, and flexible laryngoscopy showed near-complete resolution of the left vocal cord paralysis (Fig. 3).

## Discussion

A substernal goiter is thyroid tissue extending into the thoracic cavity through the thoracic inlet. About 10% are posterior mediastinal, and 90% of these occur on the right [4]. We report a left-sided retrotracheal thyroid goiter in a 39-year-old male, managed with cervical collar incision, median sternotomy, and CPB.

**Figure 4:**
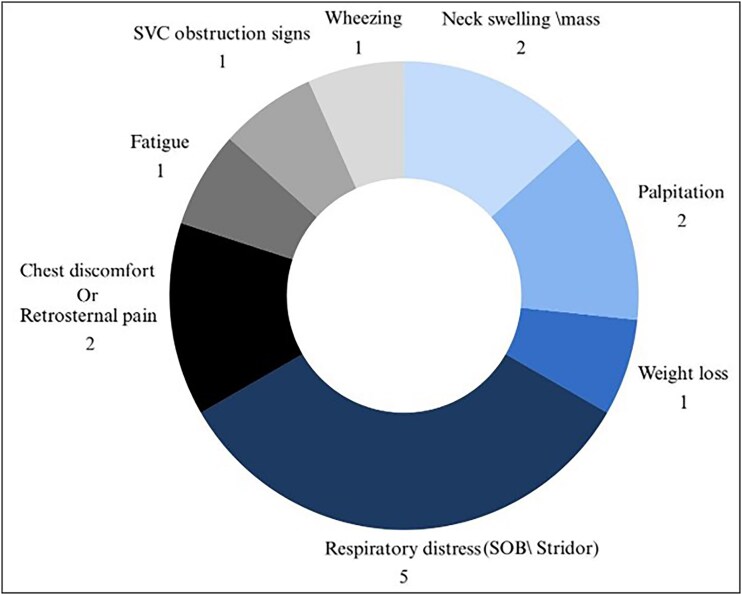
Illustration depicting the patient’s chief complaint.

Previous reports of retrotracheal thyroid goiter (Tables 1–3) include 10 cases with patients aged 47–88 years; the majority were female (8 females, 2 males) [1–3, 5–11]. Dyspnea was the most common symptom, whereas our younger male patient primarily reported palpitations and hoarseness, an atypical presentation [1–3, 5–11] (Fig. 4). Retrosternal goiters most commonly extend anteriorly, while posterior extension is relatively uncommon [3]. Posterior mediastinal involvement similar to our case has been described in a limited number of reports [3, 8, 9] (Table 1).

**Table 1:** Studies and patient characteristics.

Study ID (Last name of first co-author, year of publication)	Title	(Name of journal published in, country of origin)	(Age, gender)	Chief complaint	Thyroid function test (TSH)	Clinical size of thyroid gland and mass extension	Was a thyroidectomy performed previously?(Yes or no)	Comorbidities
(Sandasecra et al. [1])	A monster in the chest: a tale of a goiter	(Cureus, Malaysia)	(47, Male)	Anterior progressive neck swelling	Euthyroid (1.14 mIU/l) (reference range: 0.4–4.0)	Enlarged, anterior mediastinal goiter	No	NA
(Alqahtani et al. [2])	Multinodular goiter with a retropharyngeal extension: a report of two cases and literature review	(Journal of Family and Community Medicine, Saudi Arabia)	(62, Female)	Progressive neck mass	Euthyroid	Enlarged	No	Diabetes mellitus and hypertension
(Abdullah et al. [3])	Huge toxic goiter extending to the posterior mediastinum; case report with literature review	(International Journal of Surgery Case Reports, Iraq)	(70, Male)	Palpitation and weight loss	Hyperthyroidism	Enlarged, posterior mediastinal goiter	No	NA
(Nistor et al. [5])	Emergency surgical tracheal decompression in a huge retrosternal goiter	(Acta Endocrinologica (BUC), Romania)	(66, Female)	Progressive respiratory distress (inspiratory dyspnea, stridor)	Euthyroid (2.1 μU/ml) (reference range: 0.5–5.0)	Enlarged, posterior mediastinal goiter	No	Obesity and hypertension
(Ferreira et al. [6])	Total thyroidectomy by median sternotomy for treatment of substernal goiter: a case report	(Cureus, Brazil)	(61, Female)	Retrosternal burning pain and palpitations	NA	Enlarged, anterior mediastinal goiter	No	Type 2 diabetes mellitus and hypertension
(Dias et al. [7])	Acute airway obstruction due to benign multinodular goitre	(BMJ case reports, Portugal)	(88, Female)	Acute dyspnea and stridor	Hyperthyroidism 0.044 uUI/ ml (reference range: 0.4–4.0)	Enlarged	No	Aortic stenosis, systemic arterial hypertension and dyslipidemia
(Kacprzak et al. [8])	Retrosternal goiter located in the mediastinum: surgical approach and operative difficulties	(EACTS\ Interdisciplinary Cardiovascular and Thoracic Surgery, Poland)	(53, Female)	Non-specific chest discomfort	Hyperthyroidism (0.123 μl U/ml) (reference range: 0.400–4.000)	Enlarged, posterior mediastinal goiter	No	NA
(Aziret et al. [9])	An unusual recurrent bilateral posterior mediastinal goiter after subtotal thyroidectomy: case report	(International Journal of Surgery Case Reports (IJSCR), Turkey)	(61, Female)	Progressive increase dyspnea on exertion and fatigue	Euthyroid	Enlarged, recurrent bilateral posterior mediastinal goiter	Yes, bilateral subtotal cervical thyroidectomy 10 years ago	NA
(Wexler et al. [10])	Single-stage operation for giant substernal goiter with severe coronary artery disease	(Annals of Thoracic and Cardiovascular Surgery, USA)	(76, Female)	Shortness of breath and wheezing	Euthyroid (2.1 μU/ml) (reference range: 0.5–5.0)	Enlarged, substernal goiter	No	Hypertension, hyperlipidemia, diabetes, obesity, asthma, chronic tracheobronchitis, coronary artery disease (CAD), and myocardial infarction(non-ST elevation)
(Radauceanu et al. [11])	Temporary extracorporeal jugulosaphenous bypass for the peri-operative management of patients with superior vena caval obstruction: a report of three cases	(Journal of the Association of Anaesthetists of Great Britain and Ireland, UK)	(67, Female)	Respiratory distress with stridor and SVC obstruction	Recurrent thyrotoxicosis	Enlarged, retrosternal multinodular goiter	No	Rheumatoid disease, chronic obstructive pulmonary disease, and chronic schizophrenia

**Table 2:** Procedure characteristics and preoperative management.

Study ID (Last name of first co-author, year of publication)	Title	Preoperative non-surgical thyroid gland management	Preoperative non-surgical cardiac management	Preoperative investigations with findings	Surgical management	Mediastinal compression and/or deviated organs(Yes or No)
(Sandasecra et al. [1])	A monster in the chest: a tale of a goiter	No need	No	Thyroid ultrasound and CT of the neck and thorax revealed large mixed solid and cystic masses in both thyroid glands with significant mediastinal extension, causing compression of major intrathoracic veins and arteries; FNA confirmed a benign follicular nodule, and indirect laryngoscopy showed left vocal arytenoid prolapse without paralysis.	Full sternotomy and transcervical approaches with tracheostomy performed without bypass or thoracotomy	Yes
(Alqahtani et al. [2])	Multinodular goiter with a retropharyngeal extension: a report of two cases and literature review	No need	No	Contrast-enhanced CT of the neck and sestamibi parathyroid scan identified a small ectopic parathyroid adenoma medial to the submandibular gland. FNA revealed atypia of undetermined significance (Bethesda III) in the right lobe, a benign follicular nodule (Bethesda II) in the left lobe, and a right ectopic parathyroid adenoma.	Median sternotomy, and partial thymectomy mediastinal mass excision	NA
(Abdullah et al. [3])	Huge toxic goiter extending to the posterior mediastinum; case report with literature review	Two years of using antithyroid medicines	No	CT of the neck and chest revealed significant thyroid extension into the posterior mediastinum, causing tracheal and esophageal compression and deviation. The esophageal deviation was also shown in a barium study.	Collar incision (cervical incision) and sternotomy (manubriotomy)	Yes
(Nistor et al. [5])	Emergency surgical tracheal decompression in a huge retrosternal goiter	No need	No	Laryngoscopy showed normal vocal cords. X-ray revealed an enlarged mediastinal opacity with tracheal compression and right deviation. CT scan showed right thyroid lobe enlargement, left laryngo-tracheal deviation, and absence of the left thyroid lobe.	Bipolar approach (transcervical and transsternal) through a partial upper cervico-sternotomy	Yes
(Ferreira et al. [6])	Total thyroidectomy by median sternotomy for treatment of substernal goiter: a case report	NA	No	X-ray showed a large anterior mediastinal mass. CT revealed a solid, heterogeneous lesion extending from C7 to T7, reaching the right atrium.	Cervicotomy with medial partial sternotomy	NA
(Dias et al. [7])	Acute airway obstruction due to benign multinodular goitre	No need	No	CT revealed an enlarged thyroid with an extensive intrathoracic component compressing the upper trachea. X-ray showed upper mediastinal widening.	Cervical incision and sternotomy	Yes
(Kacprzak et al. [8])	Retrosternal goiter located in the mediastinum: surgical approach and operative difficulties	Iodine-131\J131 at a dose of 830 MBq	No	X-ray showed a large mediastinal mass compressing and displacing the trachea. Ultrasound revealed a massive goiter with both thyroid lobes extending beyond the sternum. CT confirmed the thyroid gland extending retrotracheally to the lower left atrium, compressing and displacing the trachea. SPECT/CT with J131 verified the mass as an extensive thyroid enlargement.	Transcervical and right thoracotomy approach	Yes
(Aziret et al. [9])	An unusual recurrent bilateral posterior mediastinal goiter after subtotal thyroidectomy: case report	NA	No	X-ray showed upper and middle mediastinal enlargement with mild right tracheal deviation and possible stenosis at the aortic arch. CT revealed a large posterior mediastinal mass extending retrotracheally from the neck.	Anteromedian sternotomy	Yes
(Wexler et al. [10])	Single-stage operation for giant substernal goiter with severe coronary artery disease	NA	No	A chest X-ray indicated a widened mediastinum, and a CT scan identified a large substernal goiter compressing the trachea and aorta.	Single-stage surgery combining thyroidectomy and coronary artery bypass grafting (CABG)	Yes
(Radauceanu et al. [11])	Temporary extracorporeal jugulosaphenous bypass for the peri-operative management of patients with superior vena caval obstruction: a report of three cases	Recurrent thyrotoxicosis required carbimazole, Lugol’s iodine and propranolol to become euthyroid	Temporary extracorporeal jugulosaphenous bypass (novel technique involving a temporary extracorporeal venovenous shunt)	High-resolution CT of the neck and thorax revealed a large retrosternal multinodular goiter causing tracheal compression to a minimum diameter of 6 mm and significant superior vena cava (SVC) obstruction.	Only thyroidectomy, neck horizontal incision made over the anterior border of the sternocleidomastoid	Yes

**Table 3:** Procedure and postoperative characteristics.

Study ID (Last name of first co-author, year of publication)	Title	Total or partial thyroidectomy	Resected thyroid weigh (g)	Hospital stay postoperative (days)	Histopathological findings	Postoperative complications
(Sandasecra et al. [1])	A monster in the chest: a tale of a goiter	Total thyroidectomy	670	5	Benign follicular nodule (adenomatous nodules within nodular hyperplasia)	Transient hypocalcemia
(Alqahtani et al. [2])	Multinodular goiter with a retropharyngeal extension: a report of two cases and literature review	Total thyroidectomy	NA	NA	Benign follicular nodule	No complications
(Abdullah et al. [3])	Huge toxic goiter extending to the posterior mediastinum; case report with literature review	Total thyroidectomy	NA	3	Benign thyroid disease (benign thyroid enlargement with colloid degeneration)	No complications
(Nistor et al. [5])	Emergency surgical tracheal decompression in a huge retrosternal goiter	Total thyroidectomy	NA	10	Multinodular goiter with epithelium hyperplasia	No complications
(Ferreira et al. [6])	Total thyroidectomy by median sternotomy for treatment of substernal goiter: a case report	Total thyroidectomy	595	4	Multinodular goiter with cystic degeneration and dystrophic calcifications	No complications
(Dias et al. [7])	Acute airway obstruction due to benign multinodular goitre	Total thyroidectomy	202	2	Benign multinodular goitre with hyperplasia	Died as a result of ventricular fibrillation
(Kacprzak et al. [8])	Retrosternal goiter located in the mediastinum: surgical approach and operative difficulties	Total thyroidectomy	NA	7	Typical goiter	No complications
(Aziret et al. [9])	An unusual recurrent bilateral posterior mediastinal goiter after subtotal thyroidectomy: case report	Completion thyroidectomy	NA	11	Nodular thyroid goiter	No complications
(Wexler et al. [10])	Single-stage operation for giant substernal goiter with severe coronary artery disease	Total thyroidectomy	NA	9	Multinodular goiter with calcification	No complications
(Radauceanu et al. [11])	Temporary extracorporeal jugulosaphenous bypass for the peri-operative management of patients with superior vena caval obstruction: a report of three cases	Total thyroidectomy	NA	2	Multinodular goiter	No complications

Preoperative investigations varied [1–3, 5–11]. CT was the most common imaging modality for substernal and retrotracheal thyroid extension [1–3, 5–11]. Several studies combined CT with thyroid ultrasound and fine-needle aspiration to assess benign nodules and associated airway or esophageal compression [2]. Additional investigations included barium swallow for esophageal deviation [3], chest X-ray demonstrating mediastinal widening and/or tracheal displacement [5–10] (Table 2).

Among 10 reported cases, various surgical approaches were used, with tracheostomy or cervicotomy as an adjunct in two cases [1–3, 5–11]. Mediastinal compression or deviation of adjacent organs was documented in eight cases, unreported in two. Despite significant airway and vascular compression, none of the previously reported cases utilized CPB. To our knowledge, our case represents the first reported use of CPB to facilitate safe resection of a retrotracheal substernal goiter (Table 2).

All reviewed cases involved total thyroidectomy [1–3, 5–11]. Postoperative hospital stay ranged from 2 to 11 days. Histopathological analysis most commonly demonstrated benign follicular nodular disease, consistent with our findings [1, 2] (Table 3).

## Conclusion

This case report describes successful management of a rare left-sided retrotracheal thyroid goiter in a 39-year-old male using cervical incision, median sternotomy, and CPB. CPB ensured hemodynamic stability. The case highlights a multidisciplinary approach and the need to refine preoperative assessment and establish guidelines for complex retrosternal and retrotracheal goiters.

### Study strengths

This case describes a rare left-sided retrotracheal substernal thyroid goiter. Strengths include CPB use for hemodynamic stability and a multidisciplinary approach with ENT, cardiothoracic surgery, and anesthesia.

### Limitations

This case is limited by incomplete clinical details. Some literature lacked procedural or long-term outcome data. Selection and reporting bias remain possible. Deviations from standard thyroidectomy—median sternotomy and unplanned CPB—were necessary due to anatomical complexity and intraoperative instability, limiting direct comparison with conventional approaches.
